# Supplementary material for: Neuropilin-1 functions as a VEGFR2 co-receptor to guide developmental angiogenesis independent of ligand binding
Source: eLife. 2014 Sep 22;3:e03720. doi: 10.7554/eLife.03720 (PMC4197402; doi:10.7554/eLife.03720)
Supplement: Supplementary file 1. — Primers used for generating, genotyping, and sequencing the Nrp1VEGF- knock-in mouse line. DOI: http://dx.doi.org/10.7554/eLife.03720.017 [file elife03720s001.pdf]

The primers used for generating the D320K expression construct are as follows:		
Primer name	Primer sequence	
outside fw	5’-CCGGCTGGAGATCTGGGATGGATT-3’	
outside rev	5’-CTGGTCACCAGGCGGATGTTTTCTG-3’	
middle fw	5’-TGGACACCAGGAGAG**AAG**TCCTACAG-3’	mutated bases in bold
middle rev	5’-CTGTAGGA**CTT**CTCTCCTGGTGTCCA-3’	mutated bases in bold
The following primers were used to amplify the short arm and clone it into the vector:		
Primer name	Primer sequence	
5’ primer	atg**gaattc**TCATGGAAGAGAAGATGTGGC	EcoRI site added in bold
3’ primer	atg**gttaac**GTATGTAGGGAGTTGAAGGGCATC	HpaI site added in bold
The primers used to amplify these homology arms are as follows:		
Primer name	Primer sequence	
5’ homology arm primer #1	atg**gcggccgc**TGTACTTTTGCTCATGAAGCCAT	NotI site added in bold
5’ homology arm primer #2	atg**gtcgac**TGCAGAAATTCTGTCTATAGTG	SalI site added in bold
3’ homology arm primer #1	atc**gtcgac**GCCTGGATTTCTTCTGCAAGT	SalI site added in bold
3’ homology arm primer #2	atc**ctcgag**ATGGGGTGGTAGGGCAGGGG	XhoI site added in bold
The D320K mutation was introduced using PCR with the following primers:		

Primer name	Primer sequence	
5' outside primer endogenous BglII site	GGATCAAATGCAGATCTTAATAC	
3' outside primer endogenous BstEII site	TACACAAAGATCTCAAACACACA	
5' mutation primer	GACTCCAGGAGAA**A**A**G**TCCTACAA	mismatch in bold
3' mutation primer	TTGTAGGA**C**T**T**TTCTCCTGGAGTC	mismatch in bold
Npn1^{VEGF-} mice genotyping primers:		
Primer name	Primer sequence	
fw	5'-TACAAAAGTGGCTTAAGGGAGA-3'	wild-type band is 305 bp<br>targeted allele is 350 bp
rev	5'-GATTGCCAGTGTGATAGGATT-3'	
Mutation site sequencing primers:		
Primer name	Primer sequence	
	5'-GGATCAAATGCAGATCTTAATAC -3'	
	5'-TACACAAAGATCTCAAACACACA-3'	
